# Supplementary material for: Proteomic Analyses of the G Protein-Coupled Estrogen Receptor GPER1 Reveal Constitutive Links to Endoplasmic Reticulum, Glycosylation, Trafficking, and Calcium Signaling
Source: Cells. 2023 Nov 3;12(21):2571. doi: 10.3390/cells12212571 (PMC10650109; doi:10.3390/cells12212571)
Supplement: Supplementary file 1 [file cells-12-02571-s001.zip › Supplementary Materials.pdf]

Supplementary Materials

Ahmadian Elmi et al.

- 4 Supplementary Figures (Figures S1-S4), included here with their legends.

- 3 Supplementary Tables (Tables S1-S3), available as separate Excel files:
    - Table S1: Complete data of the proximity-labelling/MS experiment.
    - Table S2: Complete data of the IP-MS experiment.
    - Table S3: Top hits of the proximity-labelling/MS and IP-MS experiments and the 73 proteins common to both (corresponds to Venn diagram of Figure 6B).

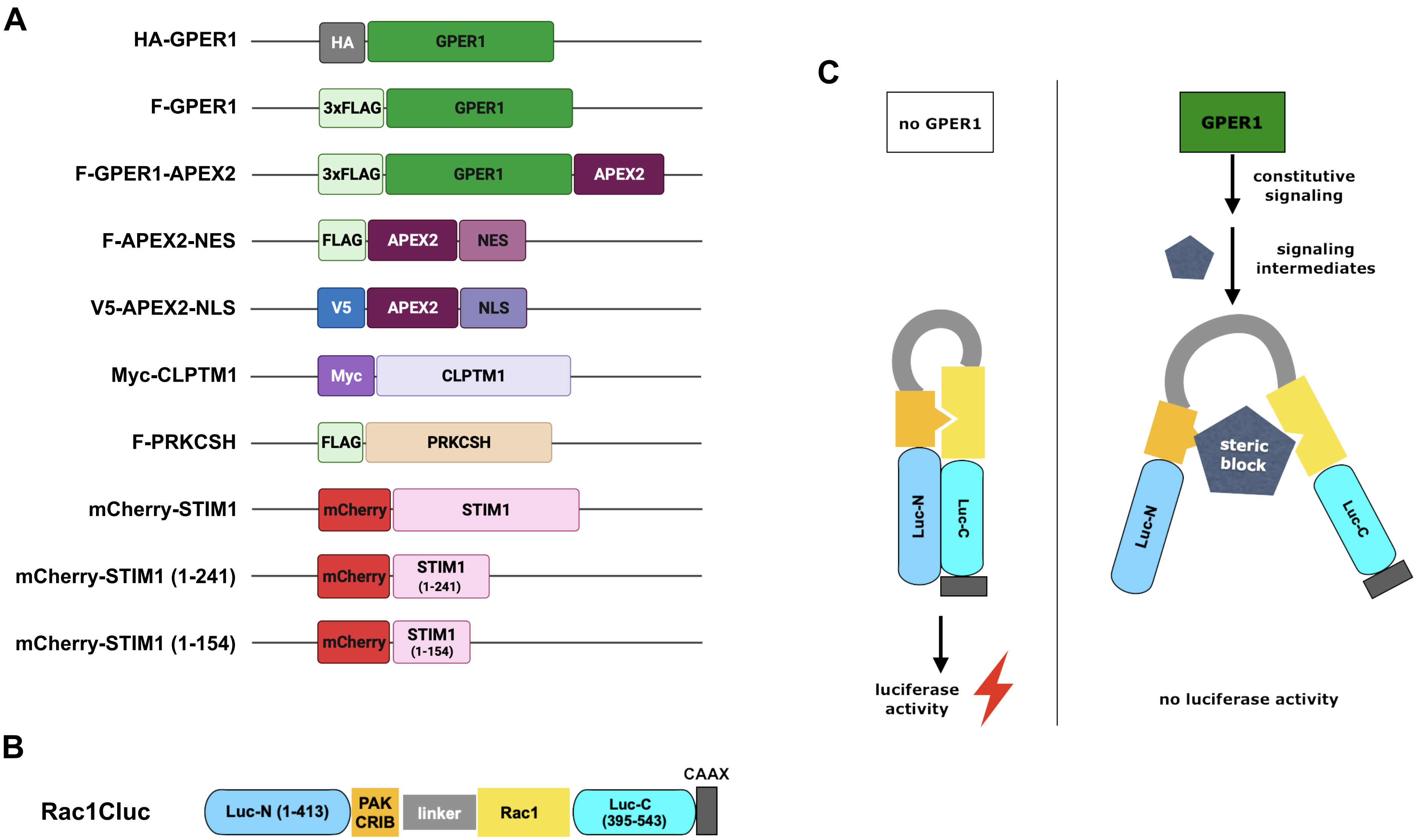
A
HA-GPER1
HA
GPER1
F-GPER1
3xFLAG
GPER1
F-GPER1-APEX2
3xFLAG
GPER1
APEX2
F-APEX2-NES
FLAG
APEX2
NES
V5-APEX2-NLS
V5
APEX2
NLS
Myc-CLPTM1
Myc
CLPTM1
F-PRKCSH
FLAG
PRKCSH
mCherry-STIM1
mCherry
STIM1
mCherry-STIM1 (1-241)
mCherry
STIM1 (1-241)
mCherry-STIM1 (1-154)
mCherry
STIM1 (1-154)
B
Rac1Cluc
Luc-N (1-413)
PAK CRIB
linker
Rac1
Luc-C (395-543)
CAAX
C
no GPER1
Luc-N
Luc-C
luciferase activity
GPER1
constitutive signaling
signaling intermediates
steric block
Luc-N
Luc-C
no luciferase activity

Figure S1. Schematic representation of plasmids and Rac1 assay. (**A**) Plasmids used in this study. Only the protein coding portions are shown in detail. The illustration was created with BioRender.com. (**B**) Scheme of the hybrid Rac1 sensor protein encoded by plasmid Rac1Cluc (adapted from). Luc-N and Luc-C denote N- and C-terminal domains of click beetle luciferase; PAK CRIB, Cdc42/Rac-interactive binding domain of p21-activated kinase; CAAX, C-terminal sequence for membrane anchoring. (**C**) Hypothetical mechanism of how the Rac1 sensor may report on GPER1 activity (inspired by).

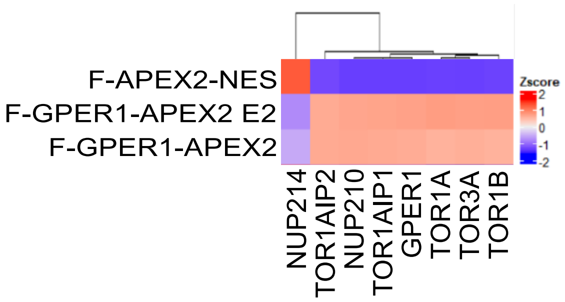

Figure S2. Nucleoporins and torsins. Hierarchically clustered heat map illustrating the enrichment of certain biotinylated proteins in the proximity-labelling MS experiment in the presence of the APEX2 fusion proteins and E2 as indicated on the left. The heat map was generated with the average Log_2 LFQ intensities of 3 biological replicates.

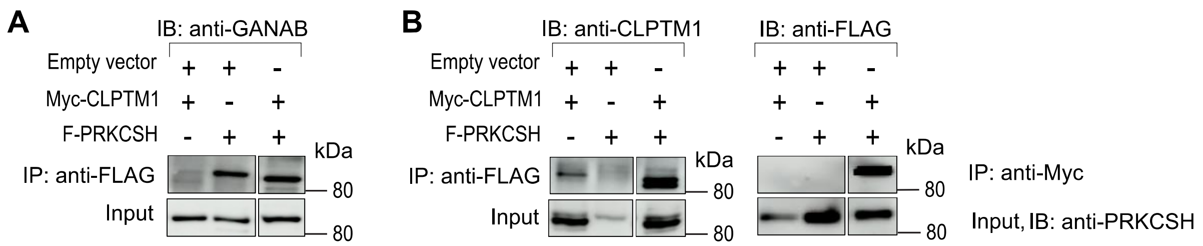

Figure S3. PRKCSH interacts with GANAB and CLPTM1. (**A**) Co-IP experiment with HEK293T cells transiently expressing indicated proteins demonstrating interaction of exogenously expressed F-PRKCSH with endogenous GANAB, independently of CLPTM1. (**B**) Co-IP experiment with HEK293T cells transiently expressing indicated proteins demonstrating interaction of exogenously expressed F-PRKCSH and Myc-CLPTM1. Numbers on the right point out molecular weights of marker proteins closeby.

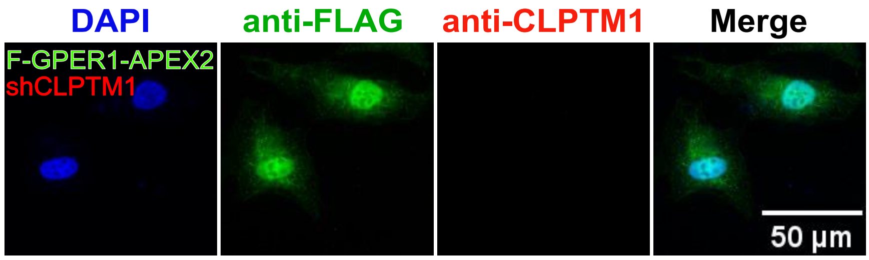

Figure S4. Impact of CLPTM1 knockdown on subcellular localization of F-GPER1-APEX2. IF experiment with Hela cells that were transiently transfected to express F-GPER1-APEX2 and with a shCLPTM1 construct that allows the production of shRNA to knock down endogenous CLPTM1 expression. Scale bar = 50 µM.
